# 598. A 3-Year Evaluation of Antibiotic Resistance Patterns in Gram-Negative Genitourinary Tract Infections Treated in Outpatient Infusion Centers (POICs)

**DOI:** 10.1093/ofid/ofab466.796

**Published:** 2021-12-04

**Authors:** Richard L Hengel, Brian S Metzger, H Barry Baker, John S Adams, Ramesh V Nathan, Rishi Bhattacharyya, Richard M Mandel, Kimberly A Couch, Claudia P Schroeder, Lucinda J Van Anglen

**Affiliations:** 1 Atlanta ID Group, Atlanta, GA; 2 Austin Infectious Disease Consultants, Austin, TX; 3 Infectious Disease Physicians, Miami, FL; 4 Knoxville Infectious Disease Consultants, Knoxville, TN; 5 Mazur, Statner, Dutta, Nathan, PC, Thousand Oaks, CA; 6 Infectious Diseases Associates, Sarasota, FL; 7 Southern Arizona Infectious Disease Specialists, PLC, Tucson, AZ; 8 Healix Infusion Therapy, Sugar Land, TX

## Abstract

**Background:**

Resistant Gram-negative pathogens (GNP) are common causes of genitourinary tract infections (GUI) often requiring outpatient parenteral antibiotic therapy (OPAT). Data are sparse regarding antibiotic resistance of GNP in patients (pts) treated with OPAT. We analyzed GNP of GUI pts treated in Infectious Disease OICs over a 3-year period stratified by location prior to OPAT.

**Methods:**

Records from 18 POICs were queried for GUI pts with ≥1 GNP receiving OPAT from 2018 to 2020. Demographics, pt location prior to OPAT, infection type, year of therapy, and GNP were recorded. Antibiotic resistance patterns were defined as extended-spectrum beta-lactamase (ESBL) or multi-drug resistant (MDR). Chi Square and Fisher’s exact test were used to determine if ESBL status was associated with GNP or location prior to OPAT (hospital vs. community). The Cochran-Armitage test was used to analyze temporal trend in ESBL expression. Statistical significance was defined as P< 0.05 for all tests.

**Results:**

A total of 634 GNP were identified in 601 pts (mean age: 64±16, 58% female). Infections were 75% complicated urinary tract infection, 20% pyelonephritis, and 5% prostatitis/other. Overall, 56% (n=339) were treated directly from the community and 44% (n=262) following hospital discharge. GNP isolated were 56% *E. coli*, 19% Pseudomonas spp., 16% Klebsiella spp. and 9% others. Of the 611 GNP with potential to express ESBL, 43% (n=265) were ESBL producers (Table 1). Significantly more ESBL-producing GNP occurred in pts discharged from a hospital prior to OPAT compared to the community (53% vs. 36%, *P*< 0.001). Overall, the incidence of MDR constituted 36% (n=231) of GNP, which did not differ by location prior to OPAT. Evaluation of ESBL incidence by year showed a significant increase from 2018 to 2020 (*P*=0.03). Although a slight increase in MDR was noted from 2018 to 2020, this was not significant (Figure 1).

Table 1. Frequency of ESBL and MDR by Location prior to OPAT

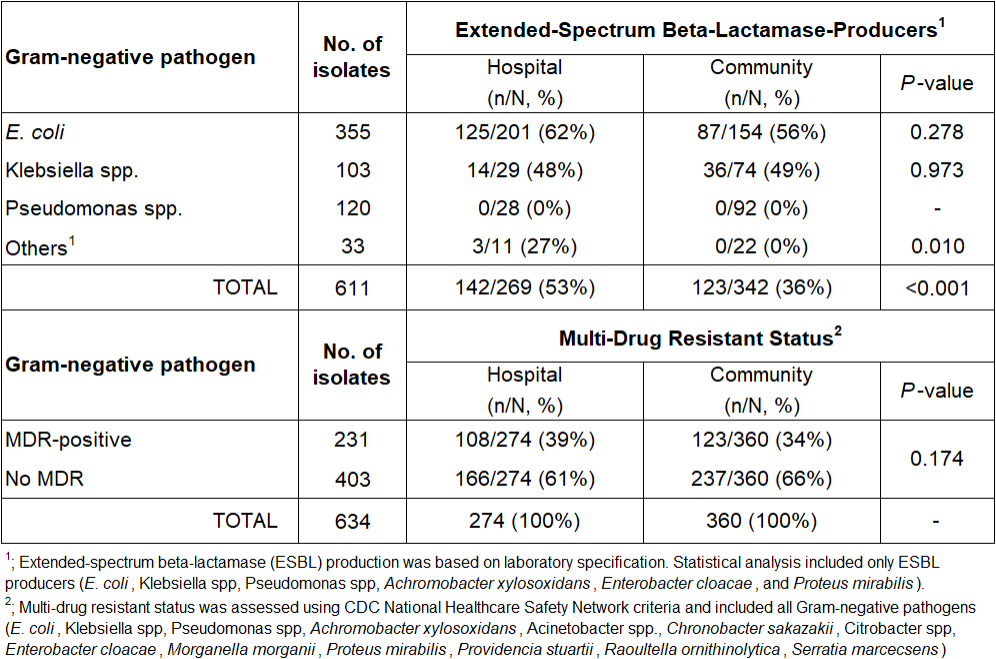

Figure 2. Prevalence of ESBL producers and MDR Pathogens by Year

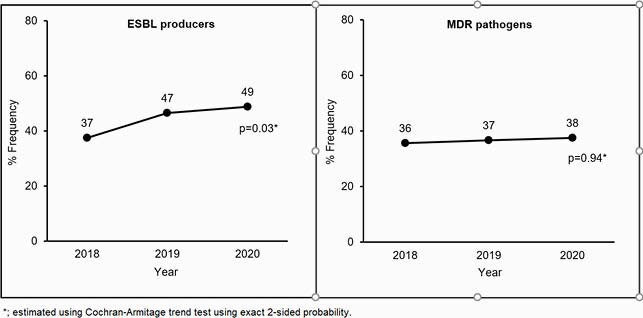

**Conclusion:**

Resistant GNP were observed in the OPAT setting for GUI with both ESBL and MDR pathogens. We saw a significantly higher rate of ESBL with GNP from hospital discharged pts compared to community-acquired infections and an increase in the overall incidence of ESBL over time. Management of Gram-negative genitourinary infections in the OPAT setting requires close monitoring of emerging resistance patterns.

**Disclosures:**

**Kimberly A. Couch, PharmD, MA, FIDSA, FASHP**, **AbbVie** (Speaker's Bureau) **Lucinda J. Van Anglen, PharmD**, **Merck & Co.** (Research Grant or Support)

